# Identification and Cloning of Differentially Expressed SOUL and ELIP Genes in Saffron Stigmas Using a Subtractive Hybridization Approach

**DOI:** 10.1371/journal.pone.0168736

**Published:** 2016-12-28

**Authors:** Oussama Ahrazem, Javier Argandoña, Raquel Castillo, Ángela Rubio-Moraga, Lourdes Gómez-Gómez

**Affiliations:** 1 Instituto Botánico, Departamento de Ciencia y Tecnología Agroforestal y Genética, Facultad de Farmacia, Universidad de Castilla-La Mancha, Albacete, Spain; 2 Facultad de Ciencias Ambientales y Bioquímica, Universidad de Castilla-La Mancha, Toledo, Spain; 3 VITAB Laboratorios, La Gineta, Albacete, Spain; Instituto de Biologia Molecular y Celular de Plantas, SPAIN

## Abstract

Using a subtractive hybridization approach, differentially expressed genes involved in the light response in saffron stigmas were identified. Twenty-two differentially expressed transcript-derived fragments were cloned and sequenced. Two of them were highly induced by light and had sequence similarity to early inducible proteins (ELIP) and SOUL heme-binding proteins. Using these sequences, we searched for other family members expressed in saffron stigma. *ELIP* and *SOUL* are represented by small gene families in saffron, with four and five members, respectively. The expression of these genes was analyzed during the development of the stigma and in light and dark conditions. *ELIP* transcripts were detected in all the developmental stages showing much higher expression levels in the developed stigmas of saffron and all were up-regulated by light but at different levels. By contrast, only one *SOUL* gene was up-regulated by light and was highly expressed in the stigma at anthesis. Both the *ELIP* and *SOUL* genes induced by light in saffron stigmas might be associated with the structural changes affecting the chromoplast of the stigma, as a result of light exposure, which promotes the development and increases the number of plastoglobules, specialized in the recruitment of specific proteins, which enables them to act in metabolite synthesis and disposal under changing environmental conditions and developmental stages.

## Introduction

**Fig 1:**
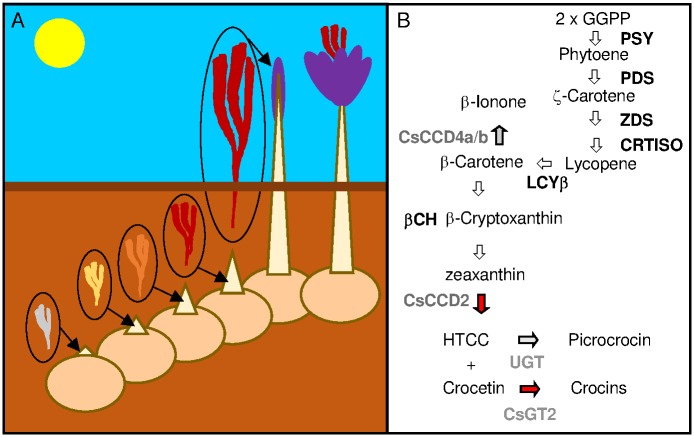
Flower development in saffron and apocarotenoids biosynthesis in the stigma tissue. (A) Drawing representing the developmental steps of flower development in saffron. At the beginning of September, one or more shoots emerge from the underground corm with the undeveloped flowers and leaves wrapped by the cataphylls, as flower develops the cataphyll grows protecting the flower up to the moment of shot emergence from the soil, which takes place during October-November, when the flower is complete developed. (B) Schematic representation of the apocarotenoid biosynthetic pathway in saffron stigma. Enzymatic reactions are represented by arrows. GGPP, geranyl geranyl diphosphate; PSY, phytoene synthase; PDS, phytoene desaturase; ZDS, Ϛ-carotene desaturase; CRTISO, carotene isomerase; LCYβ, lycopene β-cyclase; βCH, β-carotene hydroxylase; HTCC, 2,6,6-trimethyl-4-hydroxy-1-carboxaldehyde-1-cyclohexene.

The saffron stigma accumulates huge quantities of soluble apocarotenoids, crocins and picrocrocin up to the time the floral bud emerges from the soil. These components then remain relatively constant until flower senescence, when they begin to decrease [1]. These changes are transcriptionally coordinated with a parallel regulation of genes from the carotenoid pathway and with the carotenoid cleavage dioxygenase *CsCCD2* gene, involved in the formation of these apocarotenoids [2,3,4]. The synthesis of crocins takes place inside the chromoplasts from the oxidative cleavage of zeaxanthin (Fig 1) [5], and the differentiation from amyloplasts to chromoplasts is concomitant with massive crocin biosynthesis during stigma development [5]. Once synthesized, crocins pass from the chromoplast to the vacuoles where they accumulate, and chromoplast ultrastructure starts changing with an increasing accumulation of plastoglobules and empty vesicles [6]. Interestingly, the increased number of plastoglobules is accompanied by transcriptional up-regulation of another carotenoid cleavage enzyme, CsCCD4a/b, localized in the plastoglobules, and involved in the formation of a different apocarotenoid in the stigma, β-ionone, produced at high levels in preanthesis and anthesis stigmas [1,7].

Light is one of the most crucial environmental factors influencing carotenoid accumulation in plants, principally due to their role in protecting the photosynthetic apparatus against excess light [8]. The light-mediated regulation of the carotenoid pathway implies transcriptional control of genes encoding phytoene synthase (PSY) by components of the light signaling pathway as the transcription factors of the bHLH phytochrome-interacting factor (PIF) family [9,10], and the bZIP transcription factor HY5 [11]. In addition, light also increases carotenoid accumulation in chromoplast containing tissues as in some citrus species and tomato [12]. In fact, white tomato fruits that lack carotenoids are obtained by avoiding light exposure from the early stages of fruit development [13]. However, an opposite effect is observed when grapefruits are covered at the immature green stage. These exhibit an accelerated accumulation of carotenoids compared to light-exposed fruits [14], an effect also observed in certain tomato varieties [15]. An accumulation of carotenoids is also a characteristic of certain organs and subterranean tissues that accumulate carotenoids in the absence of direct light, as in the case of orange carrots or in yellow sweet potato, cassava and taro [16,17,18]. When carrot roots are illuminated, their carotenoid profile is similar to those obtained from leaves, while chromoplast development is prevented and chloroplasts are developed [17].

During the development of saffron stigmas, *CsCCD2* expression and crocin accumulation are down-regulated by light [4]. The earliest developmental stages of the flower in saffron take place beneath the soil surface (Fig 1), and during these stages crocins are accumulated at high levels in the stigma, due to the transcriptional activation of the carotenogenic genes and increased expression of *CsCCD2* [1,4]. As the flower reaches its mature stage, the expression of the carotenogenic genes is downregulated with no more net accumulation of crocins, and shortly after the fully developed flower emerges from the soil to the light (Fig 1). At this time, other apocarotenoid compounds are generated by the stigma. The production of β-ionone and other apocarotenoid volatiles seems to be induced by light in stigmas at preanthesis and anthesis [1], suggesting a complex apocarotenoid modulation by light in the development of the stigma and in carotenoid metabolism. Because of the limited molecular information concerning the regulation of apocarotenoids under different light conditions, the objective here is to improve our knowledge about the mechanisms of the transduction pathway of light-controlled apocarotenoid biosynthesis which could be helpful for programs aiming to increase the apocarotenoid content in saffron stigmas.

Suppression subtractive hybridization (SSH) is a powerful technique for the identification of differentially expressed genes, including those genes present in relatively low abundance [19]. We used SSH to isolate and characterize expressed sequence tags (ESTs) produced in red stigmas in response to light exposure during 24 hours versus stigmas submitted to 24 hours of darkness. Among the identified cDNA fragments, two of them showed the highest levels of induction by light and were chosen for further analyses and characterization. The work presented here shows the differential accumulation of several ELIP and SOUL proteins in saffron stigmas during the transition from dark to light conditions. During this transition, stigmas develop an intricate membrane network and a high accumulation of plastoglobules is observed, suggesting a function for ELIP and SOUL proteins in the reorganization of saffron chromoplasts.

## Results

### Identification of specific transcripts induced by light

Differential subtractive hybridization analysis was carried out and 22 clones were finally sequenced. Expression of these genes in light and dark treated stigmas was verified using qRT-PCR. A total of 22 clones were clearly up-regulated by light (Table 1). Several clones were associated to light responses. Clones LD3029 and LD3545 showed identity to the Arabidopsis nuclear-encoded sigma factor 5 (*SIG5*), which controls circadian rhythms of transcription of several chloroplast genes [20]; LD3750 showed identity to bZIP transcription factor Elongated Hypocotyl 5 (*HY5*), a critical positive regulator of light responses [21]; LD 3535 showed identity to HYH, predominantly involved in blue-light regulation, and whose function in part overlaps with that of HY5 [22]. LD3644 showed identity to ARF protein (Attenuate Far-Red response), which plays a positive role in phytochrome A (phyA)-mediated light signaling in Arabidopsis. LD3685 showed identity to early light-induced proteins (ELIPs), nuclear-encoded thylakoid membrane proteins that are transiently expressed immediately after light stress [23], and finally LD3572 with identity to SOUL/HBP proteins with a role in light signaling in vertebrates [24]. Among them, the expression of LD3572 and LD3685 was significantly induced by light (Table 1), and both clones were further analyzed.

**Table 1:** EST clones up-regulated by light in red saffron stigmas.

EST code	Sequence ID in the stigma transcriptome SRX848602 GSM1587351	L/D fold increase	Description	E-Value
LD3466	gnl|SRA|SRR1140761.961746.2	4,515	AT2G33690, Late embryogenesis abundant protein, group 6	2,00E-07
LD3223	gnl|SRA|SRR1767302.12929090.2	4,049	No hit	
LD3220	gnl|SRA|SRR1767302.21435612.1	4,022	No hit	
LD3535	gnl|SRA|SRR1767302.21770501.1	5,345	HYH, AT3G17609	5,00E-22
LD3572	gnl|SRA|SRR1767302.22850438.2	7,238	SOUL1, AT2G37970.1	3,00E-73
LD3684	gnl|SRA|SRR1767302.22144076.1	4,056	No hit	
LD2408	gnl|SRA|SRR1767302.21346505.2	4,251	AT3G07230, MYB-like protein	0,003
LD3773	gnl|SRA|SRR1767302.21176972.2	4,521	dihydrolipoyllysine-residue succinyltransferase component of 2-oxoglutarate dehydrogenase complex 1, AT5G55070	2,00E-33
LD2637	gnl|SRA|SRR1767302.11881954.2	4,015	No hit	
LD3787	gnl|SRA|SRR1767302.20570624.1	4,459	No hit	
LD3742	gnl|SRA|SRR1767302.22779218.2	5,207	No hit	
LD2491	gnl|SRA|SRR1767302.19318770.1	5,001	BTB/POZ domain-containing protein NPY5, AT4G37590	2,00E-32
LD3029	gnl|SRA|SRR1767302.22801613.2	4,183	SIGE AT5G24120, RNA polymerase sigma factor sigE	3,00E-25
LD2434	gnl|SRA|SRR1767302.14379052.1	4,485	No hit	
LD3685		13,910	ELIP1 P93735	2,00E-34
LD2905	gnl|SRA|SRR1767302.5420811.1	4,036	40S ribosomal protein Q0IXR7	1,00E-15
LD3658	gnl|SRA|SRR1767302.22420952.2	4,285	3'-N-debenzoyl-2'-deoxytaxol N-benzoyltransferase-like XP_010911309.1	7,00E-125
LD3018	gnl|SRA|SRR1767302.17072756.2	4,684	No hit	
LD3002	gnl|SRA:SRR1767302.21903636.2	4,048	No hit	
LD3545	gnl|SRA|SRR1767302.22144076.1	4,966	SIGE AT5G24120, RNA polymerase sigma factor sigE	2,00E-06
LD3750	gnl|SRA|SRR1767302.22918903.2	4,678	transcription factor HY5-like XP_009381123.1	9,00E-51
LD3644	gnl|SRA|SRR1767302.21610532.2	3,259	AFR Attenuated Far-Red Response XP_009387460.1	1,00E-111

### Identification of saffron ELIPs

**Fig 2:**
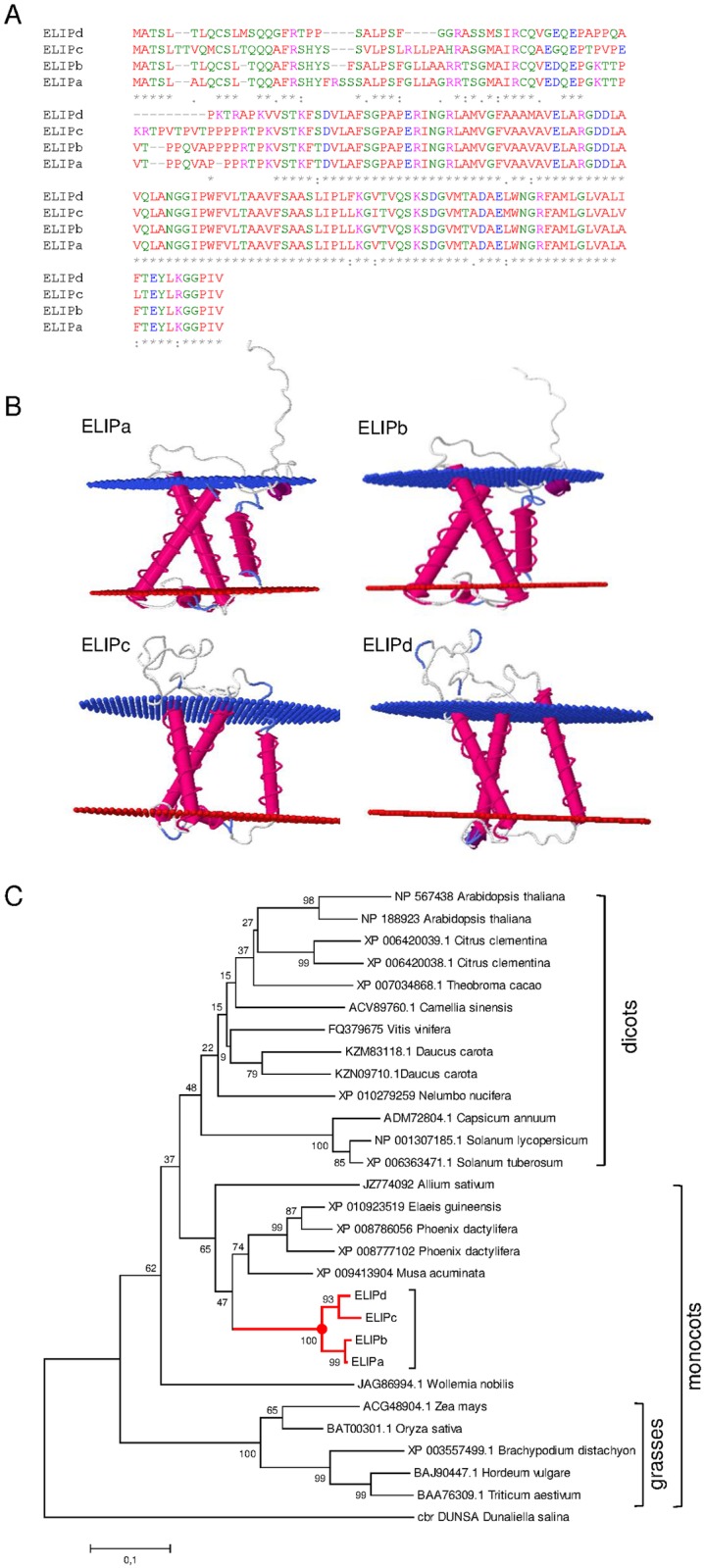
Comparison and analyses of ELIP proteins. (A) Amino acid alignment of the four ELIP proteins isolated from saffron stigmas. Conserved residues are depicted with asterisk. The colors of the residues reflect their physicochemical properties: red for small (small+ hydrophobic (incl. aromatic -Y)); blue, acid; magenta, basic—H; green, Hydroxyl + sulfhydryl + amine + G. (B) Tridimensional models of ELIPa-d and their disposition in the membrane. (C) Phylogenetic analysis of ELIP proteins. An un-rooted phylogenetic tree was constructed using MEGA6 software with the neighbor-joining method based on ClustalW multiple alignments. The percentages of replicate trees in which the associated taxa clustered together in the bootstrap test (2,500 replicates) are shown next to the branches. The sequences isolated in this study are grouped in the red subtree.

An *ELIP* cDNA fragment was identified as being highly induced by light (Table 1). Using this sequence several putative *Crocus sativus ELIP* sequences were identified in the yellow and orange stigma transcriptomes of saffron [4]. Four different full-length cDNA clones were finally identified (Genbank *ELIPa*, KX374537; *ELIPb*, KX374538; *ELIPc*, KX374539; *ELIPd*, KX374540). Both, *ELIPa* and *ELIPb* ORFs encode deduced polypeptides of 184 amino acids, with a predicted molecular mass of 19.37 kDa. *ELIPc* ORF encodes a deduced polypeptide of 188 amino acids, with a predicted molecular mass of 19.86 kDa, and *ELIPd* ORF encodes a deduced polypeptide of 172 amino acids, with a predicted molecular mass of 17.97 kDa. Main differences among the ELIP proteins were present in the N-t domain (Fig 2A). *ELIPa* and *ELIPb* shared 97.27% identity, while *ELIPc* and *ELIPd* shared 78.49% identity. All the ELIP proteins from saffron were predicted to be targeted to plastids by using the TargetP 1.1 programme (http://www.cbs.dtu.dk/services/TargetP) and to contain three similar hydrophobic domains (Fig 2B) that allowed their insertion into the membrane [25]. The three transmembrane helices of 19–23 amino acids each (Fig 2A and 2B) are also present in ELIPs from pea, barley and Arabidopsis [26]. Helices I and III of ELIPs in saffron share the Glu (E), Arg (R), and Asn (N) residues involved in binding the four core chlorophylls a in Lhcb1 [27] [28]. The second Glu (E) residue located in the C-terminal region of helix III (Fig 2A) is conserved in all known eukaryotic and prokaryotic ELIPs. Furthermore, helices I and III of ELIPs contained a conserved LAM(GAM)FAM motif (Fig 2A).

The data reported for ELIPs from pea, barley and Arabidopsis predicted that these proteins are type II membrane proteins with the N-terminus on the stromal and the C-terminus on the lumenal side of the thylacoidal membrane. The absence of chloroplasts in saffron stigmas, which only contain chromoplasts with a network of tubular membranes and vesicles inside, did not allow us to extrapolate this prediction to the ELIPs from saffron.

### Phylogenetic analysis of saffron ELIPa-d

To examine the relationship of ELIPa-d with ELIP-like proteins from other plant species, the deduced amino acid sequences were analyzed, and an un-rooted Neighbor-Joining tree was built up (Fig 2C). The relationships shown in this gene tree reflect standard groupings of monocots and dicots, with clear separation of grass-sequences from the other monocot proteins.

### Saffron ELIPs mRNA are differentially regulated during stigma development

**Fig 3:**
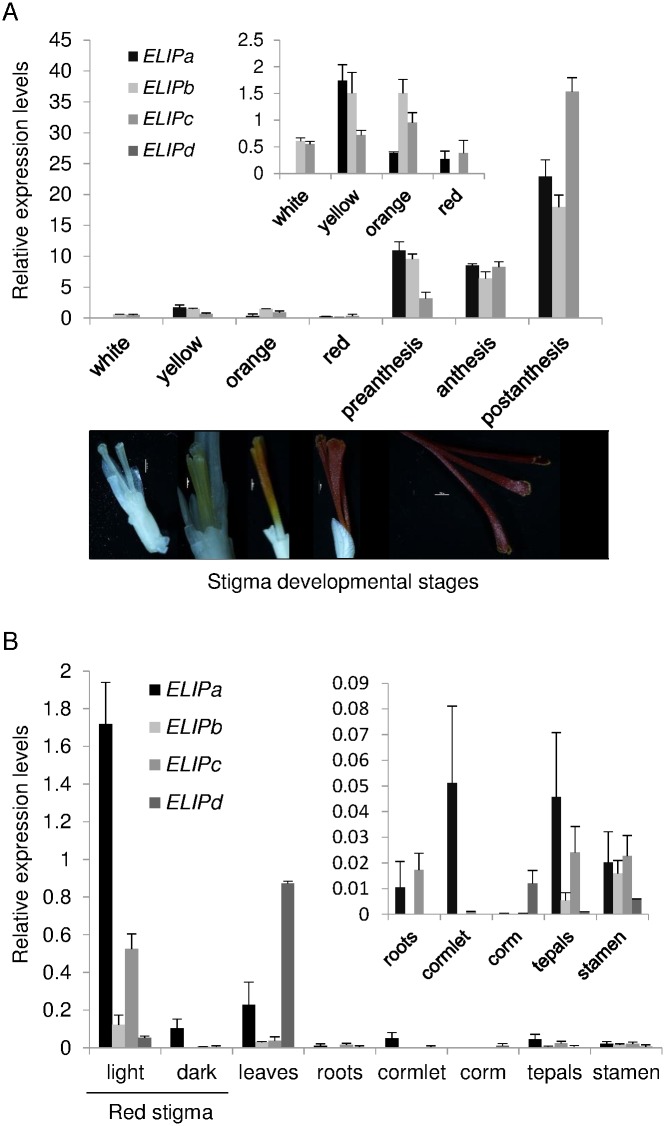
Expression analyses of *ELIP* genes isolated from saffron. (A) Relative expression levels of *ELIPa-b* during the development of the stigma in saffron. Inset are shown the relative expression levels of *ELIPa-b* in the early undeveloped stages of the stigma tissue. (B) Relative expression levels of *ELIPa-b* under two different light conditions and in several saffron tissues. Inset are shown the expression levels of these genes in root, cormlet, corm, tepal and stamen. Error bars depict SD. Below are shown the pictures of stigmas at different developmental stages: white, yellow, orange, red and stigmas at anthesis.

The presence of four ELIP proteins in saffron raised the question about their redundant physiological function. We investigated their expression levels throughout the development of the stigma (Fig 3A). All of the four *ELIP* genes showed relatively low levels of expression in the white, yellow, orange and red stigmas (Fig 3A). However, *ELIPa*, *ELIPb* and *ELIPc* showed different expression patterns during these early developmental stages. All these stages took place under the soil, in dark conditions. *ELIPa* showed an increased expression in the yellow stage and its expression decreased thereafter (Fig 3A). *ELIPb* expression was already detected in the white stage and increased in the yellow and orange stages (Fig 3A). *ELIPc* expression levels increased from the white up to the orange stage and decreased thereafter in the red stage (Fig 3A). Under the conditions tested *ELIPd* transcripts were practically undetectable in all the selected developmental stages. In the following developmental stages, from preanthesis to postanthesis the flowers had already emerged from the soil and were exposed to light. There was a marked peak of abundance of *ELIPa-c* mRNAs in the postanthesis stage (Fig 3A), with *ELIPc* being the gene with the higher expression levels.

### Expression levels in different tissues and light conditions

In general, light plays a key role in *ELIP* expression levels in plants. Therefore, we analyzed how transcripts levels of *ELIPa-d* were influenced in red stigmas that were kept in continuous light or dark conditions during 24 hours. Continuous light induced the expression of all *ELIP* genes (Fig 3B), although at very different levels, with *ELIPa* showing the highest levels of induction, followed by *ELIPc* (Fig 3B). *ELIPb* and *ELIPd* showed a lower increase, suggesting different roles of *ELIP* genes in response to light. Further, since *ELIP* genes have been shown to be induced by different abiotic stimuli in several plant species [29,30], we sought to determine whether the *ELIPa-d* transcripts can be stimulated by abiotic stresses, the expression patterns of *ELIPa-d* in response to the plant hormone ABA, hyper-osmotic stress by NaCl, heat and dehydration were evaluated by qRT-PCR (S1 Fig). Compared to the control, *ELIPa* and *ELIPc* showed the highest induction levels due to the different treatments, with *ELIPc* transcript levels being significantly up-regulated in all the conditions tested.

Expression levels of *ELIPa-d* were also analyzed in leaves, corms, cormlets, roots, tepals and stamen. In leaves, *ELIPd* showed the highest expression levels suggesting a preferred leaf-specific expression of this gene in saffron (Fig 3B). The expression levels of *ELIPa-d* in the other tissues tested were low, with an undetectable expression of *ELIPb* in subterranean organs (corm, cormlet and roots) (Fig 3B).

### SOUL proteins in saffron stigmas

**Fig 4:**
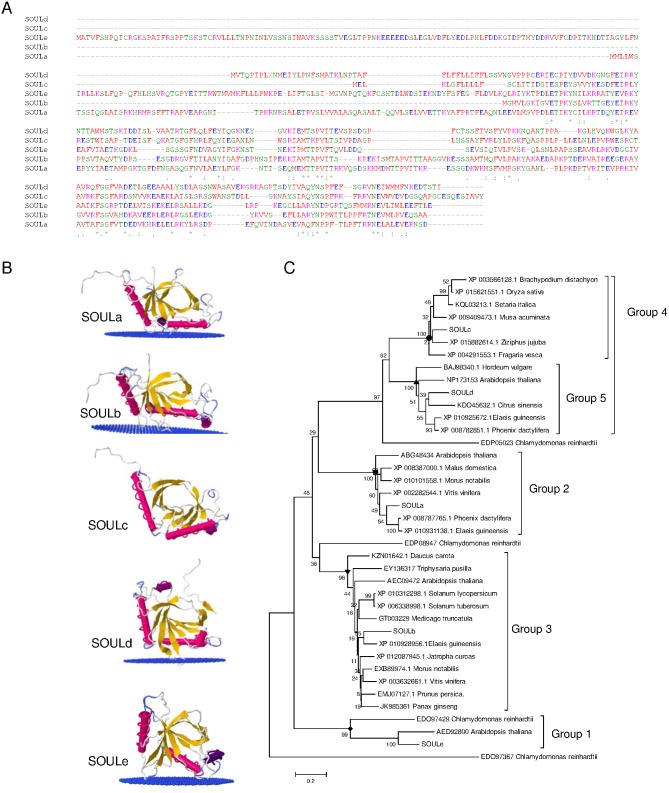
Comparison and analyses of SOUL proteins from saffron. (A) Amino acid alignment of the five SOUL proteins isolated from saffron stigmas. Conserved residues are depicted with asterisk. The colors of the residues reflect their physicochemical properties: red for small (small+ hydrophobic (incl. aromatic -Y)); blue, acid; magenta, basic—H; green, Hydroxyl + sulfhydryl + amine + G. (B) Tridimensional models of SOULa-e and their disposition in the membrane. (C) Phylogenetic analysis of SOUL proteins. An un-rooted phylogenetic tree was constructed using MEGA6 software with the neighbor-joining method based on ClustalW multiple alignments. The percentages of replicate trees in which the associated taxa clustered together in the bootstrap test (2,500 replicates) are shown next to the branches. The sequences isolated in this study are grouped in the red sub-tree.

The cDNA fragment with homology to SOUL proteins was used for the identification of *SOUL* homologues in saffron stigmas as performed for *ELIP* clones. Five *SOUL*-like genes were identified and named as *SOULa-e* (Genebank numbers: *SOULa*, KX374541; *SOULb*, KX374542; *SOULc*, KX374543; *SOULd*, KX374544; *SOULe*, KX374545) (Fig 4A). *SOULa* encodes a protein of 293 amino acids with a predicted molecular mass of 33.34 KDa. *SOULb* ORF encodes a deduced polypeptide of 206 amino acids, with a predicted molecular mass of 22.55 kDa. *SOULc* ORF encodes a polypeptide of 216 amino acids, with a predicted molecular mass of 24.45 kDA; *SOULd* ORF encodes a polypeptide of 240 amino acids, with a predicted molecular mass of 26.73 kDa, and finally *SOULe* ORF encodes a polypeptide of 388 amino acids, with a predicted molecular mass of 44.03 kDa. The SOUL proteins from saffron showed relatively low identities. SOULc and SOULd localization were assigned to secretory pathways using TargetP 1.1 and SMART. SOULa and SOULe were predicted to be localized in plastids. However, no prediction could be made for SOULb. The five SOUL proteins were modeled based on crystal structure of human SOUL protein c3r8kB [31]. The structure of all SOUL proteins consists of a central core containing stranded antiparallel β-sheets arranged in a distorted barrel, flanked by two α-helices (Fig 4B). SOULa, b, d and e seem to be able to interact with membranes with a depth/hydrophobic thickness of 4.6±0.5 Å, 7.4±0.2 Å, 3.5±0.5 Å and 5.4±0.6 Å, respectively, while SOULc seems to be soluble (Fig 4B).

Phylogenetic analysis in plants grouped the SOUL proteins into five clades based on sequence homology. Sequences in clades 1 and 2 are predicted to be localized in chloroplasts. In addition, sequences in clade 1 contained an N-terminal domain belonging to the nuclear factor 2-like superfamily (NTF2-like) present in other proteins [32]. SOULe was identified inside group 1 and SOULa was identified inside group 2. SOULb was present in clade 3, and no prediction for sub-cellular location was made for sequences from this clade. SOULc was present in clade 4 and SOULd in clade 5. Proteins in clade 5 were assigned to the secretory pathway. SOUL proteins from Arabidopsis and Chlamydomonas were also included in the phylogenetic tree for comparison and group determination.

### SOUL expression during stigma development

**Fig 5:**
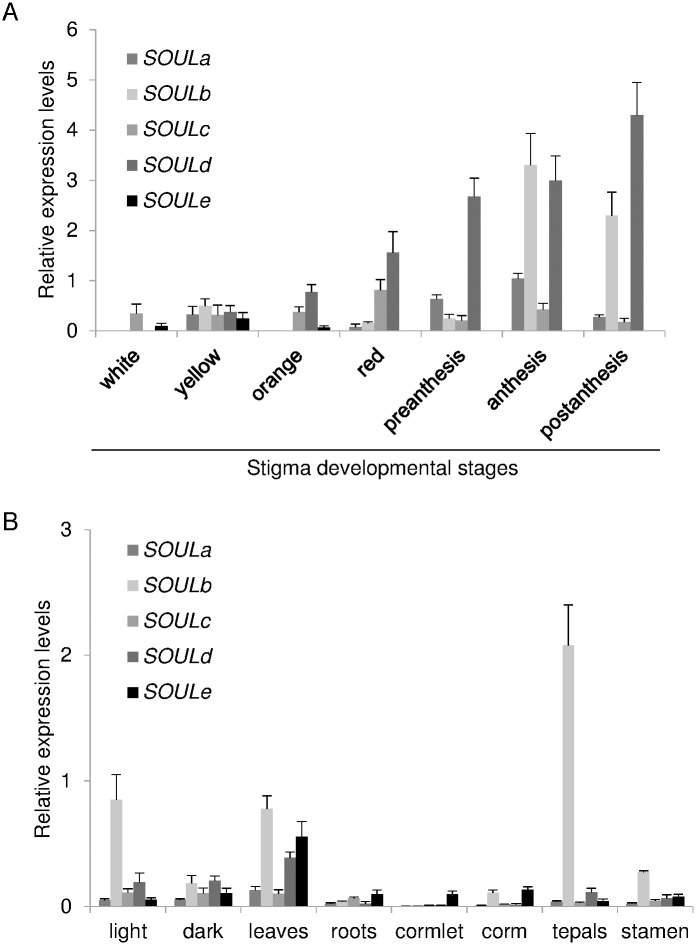
Expression analyses of *SOUL* genes in saffron stigmas and other tissues. (A) Relative expression levels of *SOULa-e* during the development of the stigma in saffron, developmental stages are the same as the ones represented in Fig 3A. (B) Relative expression levels of *SOULa-e* under two different light conditions and in several saffron tissues. Error bars depict SD.

qRT-PCR was performed in order to determine differences in the expression pattern of the identified SOUL encoding genes during the development of the stigma. *SOULa* and *b* showed a similar expression pattern, with a maximum at anthesis. *SOULa* showed a sinusoidal expression pattern, an increased expression in the yellow stage followed by a down regulation and thereafter increasing again from the red stage to the anthesis stage (Fig 5A). *SOULb* followed the same pattern although its expression levels showed more dramatic changes from the preanthesis to the anthesis stage (Fig 5A). *SOULc* showed two peaks of expression, and reached its highest expression levels in the red stage. By contrast, *SOULd* transcript levels continuously increased from the yellow stage until reaching its highest expression at the postanthesis stage (Fig 5A). *SOULe* levels were only detected in the earliest developmental stages, coincident with undeveloped flowers that remain under the soil in the dark, and the transcript levels were undetectable from preanthesis to postanthesis (Fig 5A).

The expression of *SOULa-e* was also analyzed in other tissues, and in red stigmas kept during 24 hours in continuous light or dark conditions. Overall *SOULa-d* showed their maximum expression levels in stigmas from fully developed flowers, and *SOULe* showed the highest expression levels in leaves (Fig 5B). *SOULb* exhibited high expression levels in tepals, and was also induced under light conditions (Fig 4B). However, the other *SOUL* genes were not significantly affected by the light conditions (Fig 5B). The expression levels of these genes were also tested under different stress conditions (S1 Fig).

### Expression analyses of HY5 during the development of the stigma

**Fig 6:**
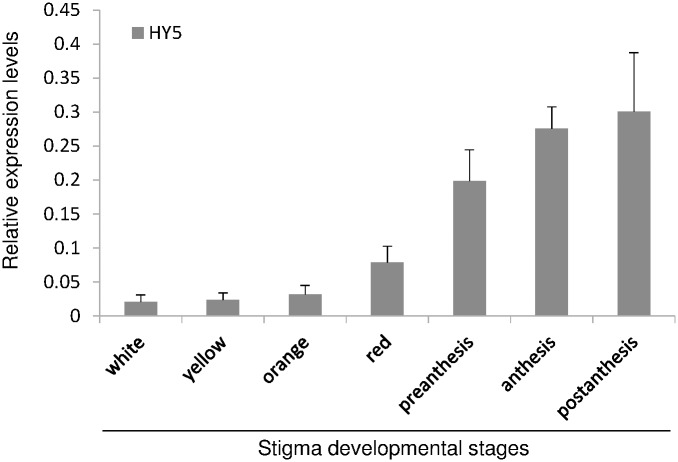
Relative expression levels of *HY5* along the development of the stigma in saffron. Developmental stages are the same as the ones represented in Fig 3A. Error bars depict SD.

It is known that the ELIP transcripts are induced by the HY5 protein in Arabidopsis [33]. The identification of homologues of both proteins in our SSH approach led us to follow up the expression of *HY5* throughout the development of the stigma in order to shed light on the relationship between this protein and the different ELIP proteins identified in saffron. The expression levels of *HY5* were very low during the initial developmental stages, from the white up to the red stages (Fig 6). However, in the following developmental stages, from preanthesis to postanthesis there was a marked increase in *HY5* expression levels (Fig 6).

## Discussion

Saffron flowers must quickly respond to changes in environmental conditions during their development. Flower initiation takes place below ground in the darkness and once all the flower parts are almost fully developed is when the elongated stalk emerges to the soil surface where the bud is ready to open [5]. Two processes associated to chromoplasts are evident during the development of the stigma. First, the accumulation of crocins in the undeveloped stigma while flowers remain under the soil in dark conditions, reaching the highest levels in the red stage [1], and second, the cessation of crocins accumulation and a rapid increase in the number of plastoglobules from the red stage onwards, when flowers are exposed to light [6,34].

Investigation of differential gene expression in red saffron stigmas kept during 24 hours in light or dark conditions may lead to the discovery of candidate genes associated to these processes in this tissue. In this study, 22 differential expression genes were identified by suppression subtractive hybridization combined with qRT-PCR. Several clones were identified as good candidates involved in light signaling and responses, including homologues to ELIP and SOUL proteins. These homologues were used to identify other members of the family in saffron and analyzed their expression throughout the development of the stigma, in different conditions and in other tissues.

### The ELIP family in saffron stigmas

ELIP are nuclear-encoded proteins present only in pro- and eukaryotic photosynthetic organisms that were first identified in pea as a chloroplast membrane-targeted product, transiently expressed, and present at the earliest stages of greening after an etiolated-to-light transition [12, 13]. At the amino acid level, members of the ELIP family are closely related to light-harvesting chlorophyll a/b-binding (Cab) antenna proteins of photosystem I and II. In higher plants they are divided into three groups, depending on the number of transmembrane helices present in their tertiary structure. With one helix are the Hlips (high light-induced proteins), Ohps (one-helix proteins), and Scps (small Cab-like proteins); Seps (stress-enhanced proteins) called also Lils (light-harvesting-like) with two helices and ELIP represented by three helices in the structure [35]. The light-induced ELIP protein identified in the suppression subtractive experiment in saffron stigmas belongs to the ELIP group. Although four genes encoding for ELIP proteins were isolated in saffron stigmas, we cannot exclude the presence of additional ELIP proteins expressed in other tissues. The number of ELIP genes is quite variable among plant species (https://phytozome.jgi.doe.gov). In the genome of *Eucalyptus grandis*, *Boea hygrometrica*, *Physcomitrella patents* and *Populus trichocarpa* between thirteen and eighteen ELIP genes are present, [36] [37], fewer are present in *Rhododendron catawbiense*, *Oryza sativa*, *Fragaria vesca*, *Brassica rapa* and *Solanum lycopersicum*, ranging from two to seven identified *ELIP* genes [38,39]. In the *S*. *tuberosum* genome only a single *ELIP* gene has been reported [40] as well as in the *Musa acumunita*, vitis, pea and tobacco genomes.

In plants and algae, isolated ELIPs show very unusual pigment-binding characteristics and pigment composition, with an extremely high lutein content when compared with other chlorophyll-binding proteins [41]. It is believed that these proteins fulfill a photo-protective role under light stress conditions. In saffron, all ELIP genes were induced by light but at very different levels. *ELIPa* showed the highest induction under light conditions followed by *ELIPc*. Both genes showed high expression levels in full-developed stigmas, and their expression was clearly increased in postanthesis. This last behavior is also followed by *ELIPb*. *CsNCED*, a gene encoding for a key enzyme involved in ABA biosynthesis in saffron [42], showed the highest levels of expression in stigmas at postanthesis, suggesting a correlation of *ELIPa*, *b* and *c* expression with stigma senescence, controlled in *Crocus* flowers by ABA. In fact, *ELIPa* and *c* expression levels increased by different abiotic stresses and *ELIPc* transcripts were clearly induced by ABA. In other plant species, *ELIP* genes have been shown to be up-regulated in responses to ABA [43], and in tobacco and pea it has been reported that *ELIP* was strongly up regulated during senescence [44,45].

Most of the studies on ELIP have been carried out in photosynthetic tissues, and much less is known about the role of ELIP in non-photosynthetic tissues. In tomato, ELIP has been suspected to play a role in chromoplastogenesis due to an increased expression during the transition from chloroplast to chromoplast, and its abundance in flowers containing yellow chromoplasts [39]. In citrus *ELIP* transcript levels are relatively low until the breaker stage, when the expression levels increase dramatically, before peaking at the ripe stage and declining thereafter. During this transition, the degradation of chlorophyll is concomitant with carotenoid biosynthesis and sequestration [46]. A carotene biosynthesis related gene (cbr) from a unicellular green algae *Dunaliella bardawil*, can be induced to accumulate massive amounts of β-carotene under light-stress conditions. Cbr was shown to accumulate coordinately with carotenogenesis induction, suggesting a function as pigment-binding protein, acting as a deposit of newly formed carotenoids [47,48]. These findings suggest the involvement of ELIP in carotenoid sequestration in plastoglobules and positively associated with carotenoid over-accumulation in chromoplasts as a mechanism of photoprotection.

### The SOUL family in saffron

SOUL/HBP proteins were first detected in the retina and the pineal gland of chickens, suggesting a role in light signaling in vertebrates [34], but their presence in other tissues suggested additional roles including control of apoptosis, supply of oxygen to metabolically active tissues, or as oxygen sensors for transcription factors that respond to oxygen levels [35]. In green algae, SOUL proteins are associated to the regulation of the eyespot size and position [49], while in Arabidopsis, SOUL proteins are involved in phytochrome-mediated red/far-red light responses [50] and implicated to heme oxygenase-mediated antioxidant pathway [51]. Moreover, it has also been proposed that SOUL proteins in plants could participate in TSPO-dependent stress responses as soluble porphyrin transporters among the different compartments of the cell [52]. SOUL proteins are encoded by a multigene family in plants (https://phytozome.jgi.doe.gov) [53]. Arabidopsis contains six SOUL encoding genes, but one seems to be a pseudogene, and four are present in Chlamydomonas. Five members that belong to the SOUL family have been isolated in this study from saffron stigmas and each gene was clustered independently in different groups as well as with different predicted locations in the cell. The most unrelated group contains the saffron SOULe, predicted to be localized in plastids and mainly expressed in leaves. SOULe appears in the same group of Chlamydomonas SOUL3 and AtHBP5 heme proteins. Chlamydomonas SOUL3 has been the best studied SOUL protein in this organism, it was found in the eyespot proteome and phosphoproteome [54,55] and it was located in the pigment globule layer. SOUL3 influences in size and position of the eyespot in the cell [49]. Beside its function as a sensor for light direction and quality, the eyespot might have additional roles mainly in chloroplast function, such as the biosynthesis of prenylquinones and carotenoids [56]. It is hypothesized that the carotenoid-rich eyespot globule was originated from thylakoid membrane-associated PGs [56,57]. In the *D*. *bardawil* PG proteome, five distinct SOUL proteins have been isolated, including a homolog of *Chlamydomonas* SOUL3. In *D*. *bardawil* plastoglobuli several unique proteins have been identified including the major lipid-associated protein CGP [48,58]. The sequence of CGP reveals partial homology to SOUL heme-binding proteins and its relationship with the proteins of group 1. Proteolysis of CGP destabilizes the PGs, indicating that CGP may have a similar role to fibrillins acting as a stabilizer of the plastoglobules [59]. Further, Phylogenetic analyses showed the presence of an Arabidopsis homolog to SOUL3, AED92800, corresponding to AtHBP5, a chloroplast localized enzyme that participates in an antioxidant pathway that might be mediated by reaction products of heme catabolism. AtHBP5 may bind and then transport the excess heme to plastoglobules [51].

The second SOUL protein identified in saffron which was predicted to be localized in plastids, was SOULa. The SOULa homolog in Arabidopsis (ABG48434, At3g10130) has been found in the plastoglobule proteome [60]. This SOUL heme-binding protein of the plastoglobules is able to bind heme and is suggested to be involved in chlorophyll degradation processes [61]. However, *SOULa* was mainly expressed in stigmas, where no chloroplasts are present, and was developmentally regulated, suggesting a different role for SOULa in saffron which has no relationship with chlorophyll degradation. Further, the expression of *SOULa* is associated with the increased number of plastoglobules in saffron chromoplasts [6], and its expression was induced by several stresses, including drought and desiccation. Increased numbers of PGs have been related to various stress factors such as excess of light, drought, senescence, desiccation, and nutrient deficiency [34]. PGs in chromoplasts contain mostly carotenoids and enzymes responsible for carotenoid biosynthesis and metabolism but also other enzymes of unknown function [60]. Among the carotenogenic enzymes, heme serves as a prosthetic group in the Z-ISO isomerase [62] and in the carotenoid ε hydroxylases that catalyze hydroxylation of the epsilon-ring of beta, epsilon-carotenoids [63]. It is possible that SOUL proteins may transport heme to the chromoplast and to the PGs to maintain heme homeostasis.

Saffron *SOULb* was mainly expressed in stigmas at anthesis and in tepals, and was the unique *SOUL* gene from saffron to be induced by light in stigmas. The chlamydomonas homolog SOUL2 was identified in a recent work from the proteomic analyses of the intact eyespot of Chlamydomonas [64], although its role has not been determined. The eyespot located in the chloroplast is composed of photoreceptor proteins and red to orange carotenoid pigments, allowing the detection of light directionality. A *SOULb* homologue has also been identified in tomato, and was differentially expressed during fruit maturation, with higher levels of expression in the ripe stage [65].

*SOULc* from saffron showed in general the lower expression levels among the other *SOUL* genes, with the highest expression in the red stigma. Interestingly, *AtHBP2* (AEC09472, At2g37970), the *SOULc* homolog in Arabidopsis, was initially identified as a phytochrome A-induced transcript that rapidly responded to light during de-etiolation [50]. However, *SOULc* was not induced by light under our experimental conditions. Takahashi et al. [66] showed that AtHBP2 bound porphyrins, including heme, and they proposed that it was a tetrapyrrole-carrier protein. This protein is supposed to be in the cytosol where it can transport heme among cellular organelles. The same function could be suggested for SOULd. The expression of *SOULd* was mainly detected in saffron stigmas, and its expression increased during the development of the stigma, reaching the highest levels after anthesis, but was not induced by light. SOULd is predicted to be involved in the secretory pathway, as its Arabidopsis homolog AtHBP1. In pepper a *SOUld* homolog was identified as a secreted protein in fruit ripening [67]. In addition, the xSOUL protein from Xenopus, containing a cleavable signal peptide, was detected as a secreted protein, and suggested to be involved in cell-cell communication [68], or might function on the cell surface as a light or redox sensor by means of the heme ligand.

### A link between ELIP and SOUL through HY5

Light is a key factor controlling plant development and acclimation processes, by regulating growth and adapting to environmental conditions plant organelles and the nucleus communicate with each other. Plastid-to-nucleus communication is of particular importance during plant stress responses associated with light. Under our research conditions, *SOUL*, *ELIP* and *HYH* (*HY5*-homolog) and *HY5* homologues were identified as the highest induced genes by light in saffron stigmas. HY5 is an important positive regulator of light-dependent gene expression that is regulated by multiple photoreceptors. HY5 is necessary for the rapid transcription of genes during the dark-to-light transition [11] and could act as a signal transducer that links hormone and light signals [69]. Numerous studies have been shown that HY5 responds to an unknown plastid signal and is suggested to be involved in retrograde signaling pathways demonstrating a convergence between plastid and light signaling networks [70]. In Arabidopsis, HY5 promotes the light induction of Elip1 [33] and Elip2 [71], and the AtHBP2 promoter was identified as a target of HY5 [11]. Such findings suggest that in saffron ELIPa and SOULb could be targets of HY5 when stigmas pass from dark to light conditions.

Several ELIP and SOUL isoforms have been detected among the proteins from PGs proteome, in different cases a structural function has been suggested as associated to the integrity of PGs under high-light conditions. In fact, the regulation of *ELIP* and *SOUL* expression is modulated by light and other stress signals, but is also developmentally regulated. Photo-oxidative damage must be a primary stressor for *Crocus* species, as they spend a considerable amount of time below ground and when they emerge they must cope with high-light conditions. Thus, it appears that saffron has evolved a strategy of *ELIP* gene expansion to aid in its ability to protect its chromoplasts from oxidative damage. In addition, abiotic stress responses generically involve the production of reactive oxygen species (ROS) in plant cells, and heme plays important roles in ROS detoxification. SOUL proteins may act as carriers to balance and buffer the state of free heme in the plastid.

## Materials and Methods

### Plant materials

Plant tissues and stigmas at different developmental stages were obtained from *C*. *sativus* grown under field conditions in the Botanical Garden of CLM (Albacete, Spain). The tissues were frozen in liquid nitrogen and stored at -80°C until required. For stress-induced treatments, red stigmas were transferred for 6 hrs. to 24-well-plates containing 1 ml water supplemented with 100 mM NaCl or distilled water that was used as a control; and incubated under simulated field conditions (12 h light/dark cycles at 22°C/10°C). Heat stress was applied by raising the temperature to 38°C for 6 h. For the dehydration experiments the stigmas were incubated under simulated field conditions in Petri dishes on Whatman No. 3MM paper (Whatman International, Maidstone, England) and collected 24 hours later.

### Construction of the subtracted cDNA libraries

For cDNA library construction, saffron corms growing in the field were transferred during one week to growth chambers simulating field conditions (12 h light/dark cycles at 22°C/10°C). 24 h before the collection of the material, the light in one chamber was constant and in the other chamber the samples were incubated under no light. Flowers were dissected and red stigmas were used in the experiment. Total RNA was isolated from dark (control) and light-treated red stigmas using the RNeasy Plant Mini Kit (Qiagen, Germany). For PCR-select cDNA subtraction, mRNAs were purified with the Oligotex^™^ mRNA Mini Kit (Qiagen, Germany). The suppression subtractive hybridization (SSH) cDNA libraries, forward (light-treated) and reverse (dark control), were prepared using a PCR-Select cDNA Subtraction Kit (Clontech, California, USA) and following the manufacturer’s instructions. Two rounds of hybridization and PCR amplification were performed to normalize and enrich differentially expressed cDNA. The subtracted cDNA was further cloned into the pGEM-T easy vector (Promega, Madison, WI, USA) and transformed into chemically competent Stellar Escherichia coli cells (Clontech, Takara, Japan). The transformed cells were selected on LB agar plates containing ampicillin (100 μg/mL) at 37°C o/n for screening.

### Identification of cDNA inserts sizes by PCR

300 white clones were randomly selected from the plates and then incubated in liquid medium supplemented with ampicillin overnight at 37°C. The SSH cDNA clone inserts were amplified by PCR using M13F and M13R primers by 20 cycles of 94°C for 30 s, 52°C for 30 s, and 72°C for 1.5 min. A 5-μL aliquot of each PCR product was electrophoresed on 1% agarose gels. The rest of the PCR reactions were used for screening by dot-blot hybridization. Briefly, the PCR product (10 μL) was denatured by adding 1 M NaOH and 200 mM EDTA, pH 8.2, to give a final concentration of 0.4 M NaOH/10 mM EDTA. Samples were heated for 10 min at 100°C and spotted onto two identical nylon positively charged membranes (GE Healthcare Amersham^™^). The two blots were UV cross-linked and hybridized with DIG-labeled forward- and reverse-subtracted cDNA probes that were prepared using the DIG High Prime DNA Labeling and Detection Starter Kit II (Roche). Hybrids were detected with alkaline phosphatase-conjugated antibody against DIG (1:1000 dilution of anti-DIG-AP). The signals were generated by treating membranes with 1% CSPD, and exposed to an X-ray film (Fuji Biomax MR film) for varying times (e.g. 1, 6, 24 and 48 h).

### DNA sequencing and data analysis

Plasmids were sequenced using an automated DNA sequencer (ABI PRISM 3730xl, Perkin Elmer, Macrogen Inc., Seoul, Korea). Computer-aided sequence similarity searches were made with the BLAST suite of programs from the National Centre for Biotechnology Information (NCBI; http://www.ncbi.nlm.nih.gov). Motif searches were made using SignalP (http://www.cbs.dtu.dk/services/SignalP) and TMPRED (http://www.isrec.isb-sib.ch/sofware/sofware.html. The proteins were modeled using the phyre2 server (http://www.sbg.bio.ic.ac.uk/phyre2/) and the PPM server (http://opm.phar.umich.edu/server.php). ELIP proteins were modeled based on c4ri3A, the crystal structure of dccd-modified psbs from spinach. SOUL proteins were modeled based on d2gova1.

### Validation of SSH analysis by quantitative PCR

We employed quantitative PCR to verify the differences in gene expression of selected target genes identified by SSH. Primers designed for each gene are given in S1 Table. Quantitative PCR was done with a StepOne^™^ Thermal Cycler (Applied Biosystems, Foster City, California, USA) and analyzed using StepOne software v2.0 (Applied Biosystems, Foster City, California, USA). Gene changes were determined using the 2^−ΔΔCt^ method by normalizing to the 18SrRNA (first ^Δ^) and then the expression was calculated for each gene by comparison to the normalized expression registered for dark conditions (second ^Δ^).

### Phylogenetic analysis

The amino acid sequences were aligned using the BLOSUM62 matrix with the ClustalW (http://www.clustal.org) algorithm-based AlignX module from MEGA Version 6.0 (Tamura et al., 2013) (http://www.megasoftware.net/mega.html). The alignments were saved and executed by MEGA Version 6.0 to generate a Neighbour Joining Tree with bootstrapping (2000 replicates) analysis and handling gaps with pairwise deletion.

### Gene expression by quantitative reverse transcription-PCR (qRT-PCR)

Gene-specific oligonucleotides were used for the expression analyses (S1 Table). Total RNAs were isolated from *C*. *sativus* stigmas at seven developmental stages (white, yellow, orange, red, preanthesis, anthesis and postanthesis stigma) and from tepals, stamens, leaves, roots, corms and cormlets, by grinding fresh tissue in liquid nitrogen to a fine powder and extracting in 1 ml of Trizol reagent (Gibco-BRL) per 100 mg of fresh tissue weight, according to the manufacturer’s protocol. The RNA obtained was treated with RQ1 RNase-free DNase (Promega, Madison, WI, USA). First-strand cDNAs were synthesized by RT from 1–2 μg of total RNA using a first-strand cDNA synthesis kit from GE Healthcare Life Sciences (Buckinghamshire, UK) and 18mer oligo dT. The quantitative RT-PCR was carried out on cDNA from three biological replicates; reactions were set up in a final volume of 25 μl in GoTaq^®^ qPCR Master Mix (Promega, Madison, WI, USA) according to manufacturer’s instructions. The constitutively expressed 18SrRNA gene was used as a reference gene [72]. The qPCR conditions consisted in an initial denaturation at 94°C for 5 min; followed by 40 subsequent cycles of denaturation at 94°C for 20 s, annealing at 58°C for 20 s and extension at 72°C for 20 s; and finally extension at 72°C for 5 min. The assays were conducted with a StepOne^™^ Thermal Cycler (Applied Biosystems, Foster City, California, USA) and analyzed using StepOne software v2.0 (Applied Biosystems, Foster City, California, USA). DNA melt curves were created for each primer combination to confirm the presence of a single product.

## Supporting Information

S1 Fig (PPTX)

S1 Table (DOCX)

## Abbreviations

CsCCD2: *Crocus sativus* Carotenoid Cleavage Dioxygenase 2
ELIP: Early Light Induced Protein
ESTs: expressed sequence tags
LHCs: light-harvesting complex proteins
PGs: plastoglubules
SSH: Suppression Subtractive hybridization
