# Supplementary material for: Identification and Cloning of Differentially Expressed SOUL and ELIP Genes in Saffron Stigmas Using a Subtractive Hybridization Approach
Source: PLoS One. 2016 Dec 28;11(12):e0168736. doi: 10.1371/journal.pone.0168736 (PMC5193429; doi:10.1371/journal.pone.0168736)
Supplement: S1 Fig — (PPTX) [file pone.0168736.s001.pptx]

## Slide 1
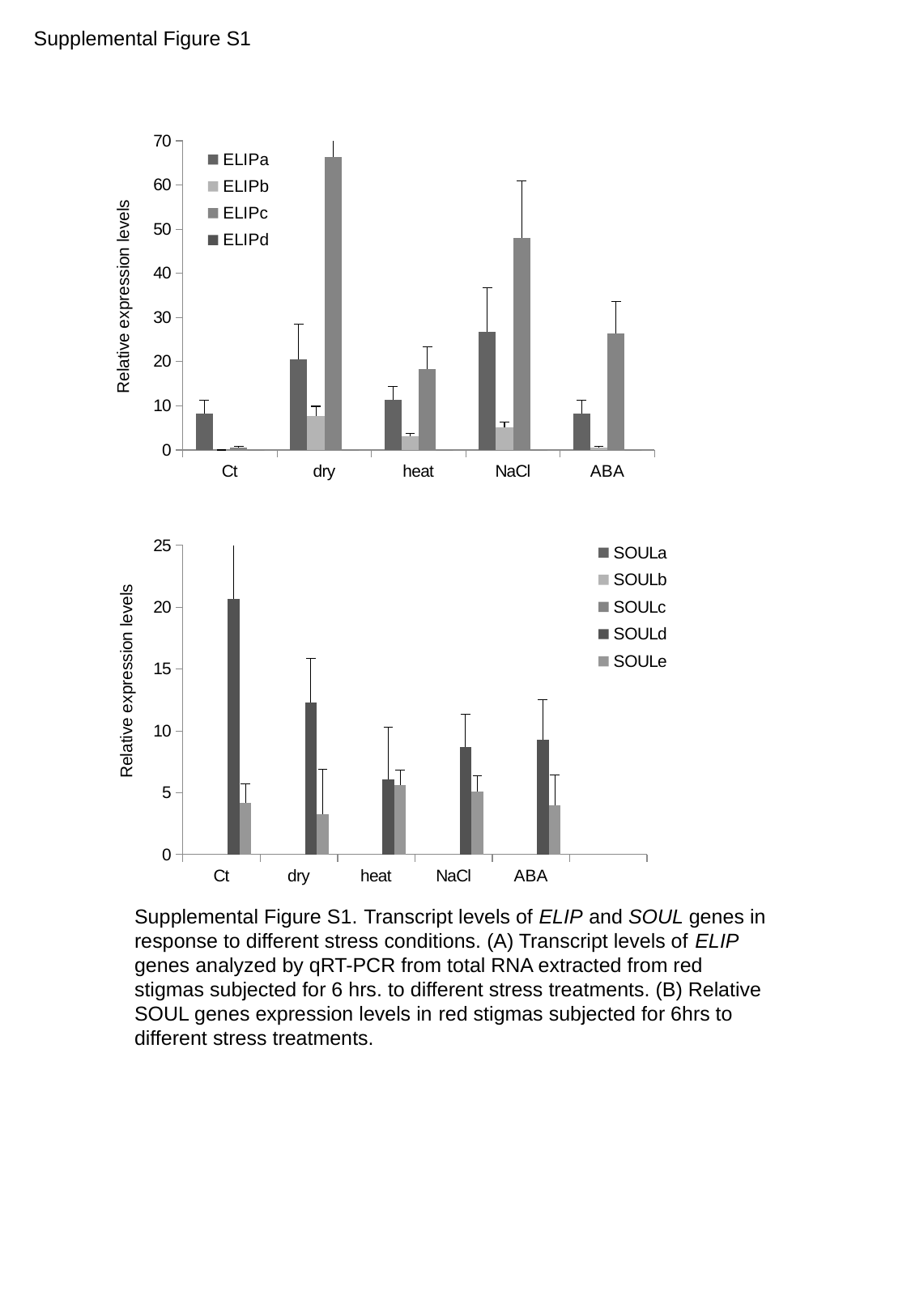

Supplemental Figure S1
### Chart
| Category | ELIPa | ELIPb | ELIPc | ELIPd |
|---|---|---|---|---|
| Ct | 8.32 | 0.0 | 0.6 | 0.0 |
| dry | 20.5 | 7.6 | 66.3 | 0.0 |
| heat | 11.3 | 3.2 | 18.4 | 0.0 |
| NaCl | 26.8 | 5.2 | 48.1 | 0.0 |
| ABA | 8.2 | 0.6 | 26.3 | 0.0 |Relative expression levels
### Chart
| Category | SOULa | SOULb | SOULc | SOULd | SOULe |
|---|---|---|---|---|---|
| Ct | 5.35 | 18.6 | 10.72 | 20.7 | 4.2 |
| dry | 7.86 | 17.8 | 8.93 | 12.3 | 3.26 |
| heat | 6.57 | 21.5 | 9.57 | 6.1 | 5.6 |
| NaCl | 8.79 | 25.2 | 10.0 | 8.7 | 5.1 |
| ABA | 6.4 | 16.0 | 11.0 | 9.3 | 3.96 |Relative expression levels
Supplemental Figure S1. Transcript levels of ELIP and SOUL genes in response to different stress conditions. (A) Transcript levels of ELIP genes analyzed by qRT-PCR from total RNA extracted from red stigmas subjected for 6 hrs. to different stress treatments. (B) Relative SOUL genes expression levels in red stigmas subjected for 6hrs to different stress treatments.
