# Supplementary material for: Identification and Cloning of Differentially Expressed SOUL and ELIP Genes in Saffron Stigmas Using a Subtractive Hybridization Approach
Source: PLoS One. 2016 Dec 28;11(12):e0168736. doi: 10.1371/journal.pone.0168736 (PMC5193429; doi:10.1371/journal.pone.0168736)
Supplement: S1 Table — (DOCX) [file pone.0168736.s002.docx]

Sequencing data

| EST code | Sequence ID in the stigma transcriptome SRX848602 GSM1587351 |
| --- | --- |
| LD3466 | gnl\|SRA\|SRR1140761.961746.2 |
| LD3223 | gnl\|SRA\|SRR1767302.12929090.2 |
| LD3220 | gnl\|SRA\|SRR1767302.21435612.1 |
| LD3535 | gnl\|SRA\|SRR1767302.21770501.1 |
| LD3572 | gnl\|SRA\|SRR1767302.22850438.2 |
| LD3684 | gnl\|SRA\|SRR1767302.22144076.1 |
| LD2408 | gnl\|SRA\|SRR1767302.21346505.2 |
| LD3773 | gnl\|SRA\|SRR1767302.21176972.2 |
| LD2637 | gnl\|SRA\|SRR1767302.11881954.2 |
| LD3787 | gnl\|SRA\|SRR1767302.20570624.1 |
| LD3742 | gnl\|SRA\|SRR1767302.22779218.2 |
| LD2491 | gnl\|SRA\|SRR1767302.19318770.1 |
| LD3029 | gnl\|SRA\|SRR1767302.22801613.2 |
| LD2434 | gnl\|SRA\|SRR1767302.14379052.1 |
| LD2905 | gnl\|SRA\|SRR1767302.5420811.1 |
| LD3658 | gnl\|SRA\|SRR1767302.22420952.2 |
| LD3018 | gnl\|SRA\|SRR1767302.17072756.2 |
| LD3002 | gnl\|SRA:SRR1767302.21903636.2 |
| LD3545 | gnl\|SRA\|SRR1767302.22144076.1 |
| LD3750 | gnl\|SRA\|SRR1767302.22918903.2 |
| LD3644 | gnl\|SRA\|SRR1767302.21610532.2 |
| Gene name | Genbank numbers |
| ELIPa | KX374537 |
| ELIPb | KX374538 |
| ELIPc | KX374539 |
| ELIPd | KX374540 |
| SOULa | KX374541 |
| SOULb | KX374542 |
| SOULc | KX374543 |
| SOULd | KX374544 |
| SOULe | KX374545 |
|  |  |
